# The Science and the Art of Surgery: Being a Treatise on Surgical Injuries, Diseases and Operations

**Published:** 1854-09

**Authors:** 


### BOOK NOTICES.

*The Science and the Art of Surgery: being a Treatise on Surgical Injuries, Diseases and Operations.* By John Erichsen, Professor of Surgery in University College, and Surgeon to University College Hospital. Edited by John H. Brinton, M.D.. Illustrated by three hundred and eleven engravings on wood. Philadelphia: Blanchard and Lea. 1854.

This is a new candidate for popular favor. We have only had time to glance over its pages; but sufficient to know that it is really what its title indicates—the Science and Art of Surgery.

In speaking of the treatment of inflammation, our author draws a judicious distinction between the sthenic and asthenic forms, while in the former he recommends venesection, purgatives, diaphoretics, calomel, &c., with the ordinary local adjuvantia, in reference to the latter he holds the following:

"In considering this part of our subject, it is of especial importance to banish the term "*antiphlogistic*," for the same treatment that would be anti-inflammatory in one form of the disease would certainly favor its progress in another. Here we must be entirely guided in the means that we adopt by the character of the symptoms. If these from the first partake of the asthenic or irritative type, we cannot at any period have recourse to the treatment that has been recommended in sthenic inflammation. If the disease commenced in an active form, the fever progressively assuming a lower and lower character merging into the asthenic and irritative conditions, so must we gradually alter the nature of our general treatment, which is always a delicate procedure, requiring much caution. Though the inflammatory fever may at first assume a sthenic form, if there is reason to believe, from the broken constitution of the patient, or from the congestive or passive character of the local inflammation, that the constitutional symptoms will not long continue of this type, we must be extremely cautious how we lower the patient by active depletion: for however high the fever may at first run, and in these cases there is often febrile disturbance of a very active character for the first few days, the disease speedily expends its force, and rapidly subsides into a low form. In such cases as these, which are of very common occurrence in London practice, more particularly in hospitals, we should never bleed, but content ourselves with clearing out the bowels, keeping the patient quiet on a moderate or low diet, and administering diaphoretic salines. As the symptoms gradually merge into the typhoid type, the pulse with increasing frequency diminishing in power, the tongue becoming dry and dark, and the other symptoms of asthenia beginning to show themselves, we must begin to give some stimulant in combination with the salines. The carbonate of ammonia, in five or ten grain doses, may be given in an effervescent form, with fifteen grains of the bicarbonate of potass, and a sufficient quantity of citric acid, every third or fourth hour. The nourishment must be increased; and small quantities of wine or alcoholic stimulants may be conjoined with it in proportion as the symptoms of debility become more and more urgent. In effecting this change, we must be careful not to run into the error of over-stimulating our patient; this may be avoided by observing the influence exercised on the pulse and tongue by the change in treatment.

"In many cases it happens that the symptoms so rapidly sink into, or, from the very first, assume so asthenic a character, that the only treatment that holds out a chance of saving the patient's life consists in the early and free administration of stimulants and mild nourishment, such as ammonia, wine, or brandy, with beef-tea and arrow-root; and of these large quantities may be required in the four-and-twenty hours, the patient evincing a tendency to sink whenever their use is interrupted. The brandy and egg mixture of the pharmacopœia, if well made, combining as it does nutriment and stimulus, is the best remedy that can be administered in many cases.

"As the asthenic passes into the irritative form, we may find it necessary to conjoin opiates in repeated doses with the general treatment. In giving these preparations, our object should be to procure sleep, and tranquillize the system, but not to narcotize the patient.

"In these low forms of inflammatory fever, congestive pneumonia, and asthenic bronchitis, usually supervene. In this complication, the following draught may be advantageously given every third or fourth hour:—℞. Tinct. camph. comp. (tinct. opii. camph. U.S.) min. 20, ammonia carbonatis. gr.v., decocti senegæ, ℥iss., with rubifacients, blisters, or dry cupping to the chest. The diarrhœa that not unfrequently occurs must be met with aromatic confection, kino or catechu in chalk mixture; and if the urine cannot be passed, it must be drawn off by the catheter."

The same rules are laid down for the treatment of gangrenous inflammation. We have used in this latter, with much success, the tr. ferri muriatis, which, as our readers will remember, has been recommended so highly in erysipelas. We have given it in doses of from twenty to thirty drops every three or four hours, until the general and local symptoms began to abate. We do not believe this to be a specific; but we have repeatedly seen, under its influence, a small, quick pulse become slow and full, the organic actions, both general and local, stimulated, the line of demarcation between the living and dead tissues rapidly formed, and the progress of the disease at once arrested.

This remedy must, of course, always be administered with a reference rather to the general condition of the patient than to the local disease; but we think that in a large number of cases, especially among hospital patients, it is indicated. Under the head of operations we find the following directions for the preparation of the patient:

"This preparation must not consist in any routine system of purging and starving, which is ill-calculated to support the constitution against the call which will be made upon its powers, but, by adopting our means, according to the condition of the patient. Indeed, in many of the more severe cases of compound fracture and disease of the joints, it is only by the use of a nutritious diet, and by the administration of stimulants, often in large quantities, that the patient can be brought into a condition to bear the shock and consequent depression of the operation. It will often be found necessary to administer from half a pint to a pint of brandy, with perhaps a considerable quantity of wine, together with eggs, beef tea, and other nourishment, for some days before the operation can be undertaken. This is more particularly the case with hospital patients of bad constitution, who have met with serious accidents, attended by much suppuration and irritative fever. In the more chronic cases, the time should be seized for the operation when the secretions are free, the tongue clean, and the action of the skin and kidneys in a healthy state—and above all, the mind should be kept tranquil and hopeful, being allowed to dwell as little as possible upon the impending event."

Professor Erichsen uses chloroform, believing that it diminishes the danger from shock, while it secures to the patient immunity from suffering. Our author's views of the mode of administration, the points to be attended to, the effects of the anæsthetic, and the conditions in which it is admissible, will be seen from the following extract:

"The *administration of chloroform* is best commenced before the patient leaves his bed. This should never be given but by a person accustomed to its use, and in whose capability the surgeon has full reliance, as nothing embarrasses him more, during the operation, than to have any doubt about the chloroform being properly administered. The following is the way in which chloroform may, I think, most conveniently and safely be administered. On a piece of folded lint, about two inches square, and consisting of three doubles, about ʒj of choloroform is poured, and the lint is then held at a distance of about three inches from the nose of the patient, so as to permit the very free admixture of air with the first few inhalations. After a lapse of about half a minute, the lint is brought nearer to the patient's nose, to within a distance of perhaps an inch, being never allowed to touch; at the same time a porous towel is laid over the face of the patient, and the hand of the operator, so as to prevent the escape of the chloroform vapor, but not to interfere with the admission of air. During the whole time it is the duty of the administrator to keep his hand on the pulse, and occasionally to examine the pupils of the patient.

"The principal points to be attended to during the inhalation of this potent agent are, that it shonld not be given too suddenly, or in too concentrated a form, and that whilst under its influence the patient be not raised up off the couch to table If the lint is too much saturated with it, and be held too closely applied to the mouth and nostrils, the patient will not be able to get sufficient atmospheric air, and may speedily become asphyxiated, choking violently, struggling to get free, and becoming purple in the face, with a full slow pulse. Whilst under its influence the patient should never be raised up, as has just been stated; for as this agent exercises a powerful sedative action on the heart, sudden and perhaps fatal syncope may ensue, by putting the patient in the erect position. Hence, also, it is dangerous to administer it in those operations that require to be performed whilst the patient is in the erect position. It is well to caution the patient not to take anything to eat for two or three hours before its administration, lest it induce vomiting of the partially digested meal. With due caution, it may be given with perfect safety to individuals of all ages. I have operated on infants less than a month old, as well as octogenarians, under its influence. In administering it to young children, Dr. Snow recommends that it should be diluted with rectified spirit. The operation should never be commenced until the patient is fully under the influence of chloroform, so that the muscles are perfectly supple and relaxed, as otherwise the semi-conscious struggle of the patient will only tend to embarrass and increase the difficulties of the operator.

"The first influence of chloroform appears to be exercised on the nervous system, the patient becoming excited and talkative; but this passes away speedily, a state of complete insensibility coming on, the muscular system at the same time becoming paralyzed, and the patient consequently being completely deprived of sense and motion. When the chloroform is given slowly and carefully, it enters the blood, and being conveyed by this fluid to the heart and nervous centres, acts directly upon these organs. Its influence upon the heart is well marked, the pulsations becoming less frequent and forcible, and are indeed often so much diminish-in power, that the jet of blood from cut arteries is materially lessened in force. The respirations become shallow and feeble in proportion as the sensibility of the nervous system and the energy of the muscular movements are lessened, and the blood becomes dark and venous; in fact, a semi-asphyxial condition sets in. When in this state, the patient is on the very verge of death, and requires the most careful watching on the part of the person who administers the chloroform. His finger should never be off the pulse, or his eyes taken away from the countenance of the patient, as the inhalation of a small additional quantity of this potent agent, the application of the vapor in too concentrated a state, or suddenly raising up the patient, may occasion a fatal syncope from paralysis of the heart.

"In certain diseased conditions of the system, the administration of chloroform is scarcely admissible; this is more particularly the case in affections of the heart and brain. In diseases of the lungs, as in case of phthisis, it may be given with perfect safety; but as it exercises, during the early stages of its administration, a tendency to determination of blood to the head, and in the more advanced stages, a decidely depressing influence upon the heart's action, we should be careful in administering it in congestive affections of the brain, or in diseased conditions of the heart, more particularly when that organ is fatty, and weak in its action. But, though these states should render us cautious in the employment of chloroform, they do not altogether contra-indicate its use; and when the patient is so irritable that it is evident that he could not bear the shock of an operation without having his sensibility blunted by this invaluable agent, it may be administered, though with great caution. In an old man, with fatty and dilated heart, whom I lately cut for stone, and on whom it would have been impossible to have operated with any chance of success without chloroform, on account of his extreme irritability, no ill effects resulted from its inhalation.

"When injurious effects have followed the administration of chloroform, they have arisen from two opposite causes, the induction of asphyxia or of syncope; both conditions being well marked by the ordinary signs attending them—the asphyxia having been induced by the vapor being administered in too concentrated a form, without admixture of atmospheric air; the syncope, by the depressing influence of the agent on the heart's action. In the first case the treatment must consist in the establishment of artificial respiration, if necessary through an opening in the trachea, dashing cold water upon the face, and exposing the surface of the body to a cold current of air. When syncope has come on, the transmission of electro-magnetic shocks through the chest, head, and spinal cord, with the establishment of artificial respiration, will be the most effectual means of restoring suspended animation."

The American editor states in this connection that chloroform is not generally employed in this country, preference being given to sulphuric ether. We presume he represents the views and practice of the profession in Philadelphia; but, unless we are very much mistaken, the proposition is not true when applied to *this country*. The editor may possibly be laboring under the mistaken idea that all our surgeons, north and south, east and west, not only think as Philadelphia thinks, but are disposed to sleep as Philadelphia sleeps.

One can hardly restrain a smile at his closing sentence: "The numerous fatal consequences, however, which have attended, and are daily resulting from the employment of chloroform in Europe, have led to the entire abandonment of its use in this country."

Our author devotes only three pages to the subject of ununited fractures. After briefly enumerating the causes of non-union, the different operations resorted to for the purpose of effecting re-union, he sums up with the following conclusions:

"On reviewing the various methods that have been recommended for the re-establishment of union between the separated fragments, it would appear that the excitation of proper inflammatory action, by the introduction of the seton, or by driving in ivory pegs, promises the most satisfactory result. It is by no means necessary to remove the fibrous band that intervenes between the fragments in cases of false joints, for if the proper amount of inflammatory action be set up, this either undergoes osseous transformation, or a sufficient quantity of callus is thrown out around it to consolidate the fracture."

It is known to the readers of this journal that Professor Brainard has recently devised a new method for the treatment of ununited fractures. This was made the subject of an essay which received the prize at the recent meeting of the American Medical Association. Having been kindly permitted by the author to see this esssay, and become acquainted with his views, we now have a case which we are treating by his method, namely, by perforating the bones with a drill. The case, with the results, will be reported in a future number of the journal.

Just as we are writing, the September number of the *New York Medical Times* has come to hand, in which we find reports of two cases of united fracture, treated in the *New York Hospital*, by Prof. Brainard's method. We copy the article entire :

" The very large number of fractures which are constantly under our care in this hospital—greater by far, I believe, than in any other institution in the country—gives to the subject of non-union of fractures a particular interest and importance to us. In the right management of this misfortune a proper appreciation of the causes which have been operating against the consolidation of the bones seems to be of great importance as a guide to us in our endeavors to bring about the wished-for union. We may explain most of the cases, I think, by reference to one of the three following circumstances :

1. *Want of power* in the system to produce or to perfect the bond of union, as is sometimes seen in exceedingly old, enfeebled patients, or in those debilitated by disease, starvation, &c.

2. *Obstructed power.*—When the vigor and plastic power of the system is complete, but when some local cause interferes with union, as too great separation of fragments, the interposition of muscles between the broken ends, too great inflammation, too much motion during the cure, &c.

3. *Diverted power*, as in those cases where the plastic powers are perfect, and the local conditions not unfavorable, but where, from the presence of some other important process, as gestation or lactation, or from severe injuries of some neighboring parts, the energies of repair are diverted from the fracture, and expended in whole or in part upon these other processes or injuries. The patient before you presents an example of the latter class. He received, thirteen months ago, from the fall of a heavy steam boiler, an injury of his arm, which not only broke the bone four inches above the elbow, but tore and lacerated almost the whole length of the fore arm, to the extent which is pretty well indicated by these cicatrices. His injuries of the fore-arm have at last healed, after a year's tedious suppurating, granulating, cicatrizing, and contracting; but, in the meantime, it would seem that nature's attention had been withdrawn entirely from the broken humors; for it remains ununited, and with scarcely a trace of any effort toward consolidation. If my view of this case is correct, you will say why will not nature, now that she has finished the work on the fore-arm, go on and accomplish the union of the arm. She does not, and she will not do it, for this reason, viz., that so much time has elapsed since the injury, that the reparative nisus has probably ceased for ever. This disposition towards reparative actions, you know, commences with and is excited by the injury done to the part, and if proper conditions are supplied, it shows itself with the certainty of a law of nature. Even if these proper conditions are withheld for a certain time, the tendency to reparation still exists, and, though postponed, will show itself in cases of fractured bones, many weeks and even months after the original injury. This fact is well illustrated in the man in the Cottage C., whose fracures remained ununited for many weeks, on account of his miserable, starved condition before he came to us. As soon, however, as he got plenty of good, nourishing food, and began to feel its influence, union commenced, and proceeded rapidly. But if the necessary conditions are too long withheld, it would seem as if the tendency to repair had become exhausted; no action takes place, though everything seems favorable for it, and this inactive condition continues indefinitely. Now, under these circumstances, which are precisely those in which I consider our patient to be, nothing will be done toward the union of this fracture unless we can re-excite this exhausted reparative condition; and this is the principle upon which the cure of these cases always is, or ought to be founded. First remove the obstacles which have been preventives of union, and then re-excite the reparative nisus. I need not recount to you the various methods in use to accomplish this purpose. You probably all are aware that the seton, resection, wiring the bones, introducing ivory pegs, &c., are all founded on the principle above mentioned, and are all of them in their proper place eminently valuable surgical resources. Statistical reports show that these methods have been followed by a great and satisfactory degree of success; but, unfortunately, this success has not been attained without danger, and in a certain proportion of the cases where these severe operations have been performed, death has been the result. It is this fact which makes us so solicitous to devise some procedure in these cases which, while it shall be effectual, shall also be safe. I propose, in this case, to adopt a plan which was first suggested to us by Dr. Brainard, of Chicago, and which consists in boring through the opposed fragments with a medium-sized drill, and thus wounding in several points the surfaces where we wish to excite the new action. These wounds can be made, all of them, through a single little hole in the skin, by sliding the skin along, and passing the drill in various directions. After these holes have been made, the small external wound is closed with adhesive plaster, and easily heals. [The operation was performed, four holes being drilled through both fragments. The bone was so soft that the drill passed with great ease The arm placed upon a tin splint.]

"June 14th.—I present you with another case of fracture which has now refused to unite, though it is three months since the leg was broken. There seems to be no good reason why this fracture should not unite; and I therefore propose to treat it in the same manner as we treated the last case, by drilling holes through the opposed fragments. [Six perforations were made through the tibia, and the limb placed in a fracture box.]

June 26.—The arm has presented very little sign of increased action; some tenderness existed round the fracture for a few days, but this has all disappeared; and I fear we shall fail here, from having too little, rather than from too much action. The leg, on the contrary, you see, has become inflamed and swollen round the seat of fracture; and, in fact, suppuration has been caused by the operation. Happily, however, this suppuration is subcutaneous, and I have and shall endeavor to keep it so. Instead, therefore, of making a free incision into this abscess, thereby converting the case into a compound fracture, I have made a very small opening, and had the matter gradually evacuated as it collected, morning and evening. Thus far, the plan has succeeded admirably. The abscess is diminishing, is free from pain, and everything promises well. [By the 19th of July the abscess, which continued to be managed in this way, had entirely healed; and on the 29th of July he was discharged, with the leg firmly united. The arm presented no evidence of union on the 1st of July. Erysipelas attacked his arm, the old scars of the fore-arm re-ulcerated, and, as the erysipelas subsided, abscess occurred about the seat of fracture; which determined us, considering the maimed condition of the fore-arm, to amputate the arm above the fracture."

The failure in the case of the arm must evidently be referred to the general condition of the patient.

We have not time to follow our author further. We can only say that the book is a good exponent of the present condition of English surgery; and as such we recommend it to our readers.

For sale by Keen and Lee, Chicago. J.

